# Interfacial polarity-driven self-assembly of organic core/shell heterostructures with directional Fabry–Pérot resonance

**DOI:** 10.1039/d5sc05873b

**Published:** 2025-09-24

**Authors:** Jin Feng, Zhen-Yu Geng, Yi Zong, Chuan-Zeng Wang, Shu-Hai Chen, Hong-Tao Lin, Li-Wei Xie, Xue-Dong Wang

**Affiliations:** a School of Chemistry and Chemical Engineering, Shandong University of Technology Zibo 255000 China linht@sdut.edu.cn; b State Key Laboratory of Bioinspired Interfacial Materials Science, Institute of Functional Nano & Soft Materials (FUNSOM), Soochow University Suzhou 215123 China wangxuedong@suda.edu.cn; c Suzhou Key Laboratory for Radiation Oncology, Department of Radiotherapy and Oncology, The Second Affiliated Hospital of Soochow University Suzhou 215004 China xlw511605617@163.com

## Abstract

Organic core/shell heterostructures (OCSHs) can exhibit diverse optical functionalities through molecular-scale energy-level modulation and interface engineering. However, the construction of structurally well-defined, optically responsive OCSHs with ordered interfaces remains a significant challenge. Here, we proposed an interfacial polarity-driven self-assembly strategy to achieve the directional construction of OCSHs comprising a charge-transfer (CT) cocrystal core and an alloy shell. Selective interfacial complexation and polarity-driven molecular coupling were achieved by sequentially introducing CT cocrystals with gradient intermolecular interaction strengths, thereby disrupting the intrinsic growth pathway of the core and triggering directional alloying of the shell. The resulting OCSHs exhibit highly ordered Fabry–Pérot (FP) cavity modes and orientation-dependent emission, enabling multistate optical logic encoding. Moreover, the approach exhibits broad applicability across multiple CT pairs, affording structurally integrated heterostructures with tunable dual-emission profiles and potential for white-light emission. This work provides a robust framework for constructing hierarchical organic photonic architectures and programmable light-manipulation systems *via* interfacial interaction engineering.

## Abstract 2

An interfacial polarity-driven self-assembly strategy enables the construction of organic core/shell heterostructures with ordered interfaces and alloyed shells, thereby generating anisotropic Fabry–Pérot resonance and optical logic outputs.

## Graphical abstract

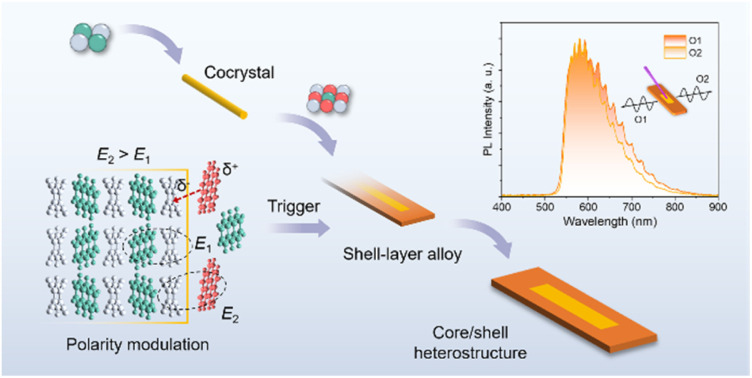

## Introduction

Amid growing interest in organic field-effect transistors (OFETs) and optical information processing, organic crystalline materials have emerged as ideal candidates for constructing functional optical devices due to the high molecular designability,^1–4,39^ tunable band structures,^5,6^ and potential for multiscale integration.^7,8^ Notably, controllable self-assembly driven by noncovalent interactions, such as charge-transfer (CT), π–π stacking, hydrogen bonding and halogen bonding, enables the bottom-up fabrication of organic micro/nano-optical devices with defined morphologies and functional heterogeneity.^9–11^ Among these architectures, organic heterostructures combine molecular components with distinct structural and electronic characteristics to achieve synergistic control over lattice organization and energy-level alignment,^12–14^ providing an ideal platform for Fabry–Pérot (FP) resonators employed in organic photonics.^40,41^ The spatial modulation of optical behavior is made possible through this structural diversity, and multifunctional integration, including wavelength conversion, exciton transport and directional waveguiding, is achieved owing to such structural characteristics.

Nevertheless, the controlled synthesis of organic heterostructures, particularly organic core/shell heterostructures (OCSHs),^15,16^ remains highly challenging. On the one hand, the nucleation and growth of multicomponent cocrystals are often limited by unbalanced intermolecular interactions, frequently resulting in phase separation, mixed crystallization, or disordered aggregation.^17–19^ On the other hand, the formation of OCSHs demands precise spatial layering and accurate energy-level alignment.^20–22^ These hierarchical configurations depend not only on thermodynamic stability but are also highly sensitive to kinetic factors including molecular competition (molecular concentration, intermolecular interactions and diffusion dynamics) and interfacial reactions during assembly.^23,24^ Therefore, achieving ordered assembly and structural stability in organic heterostructures, while preserving well-defined core/shell interfaces and effective functional coupling, remains a pressing challenge in the photonics field.

**Fig. 1:**
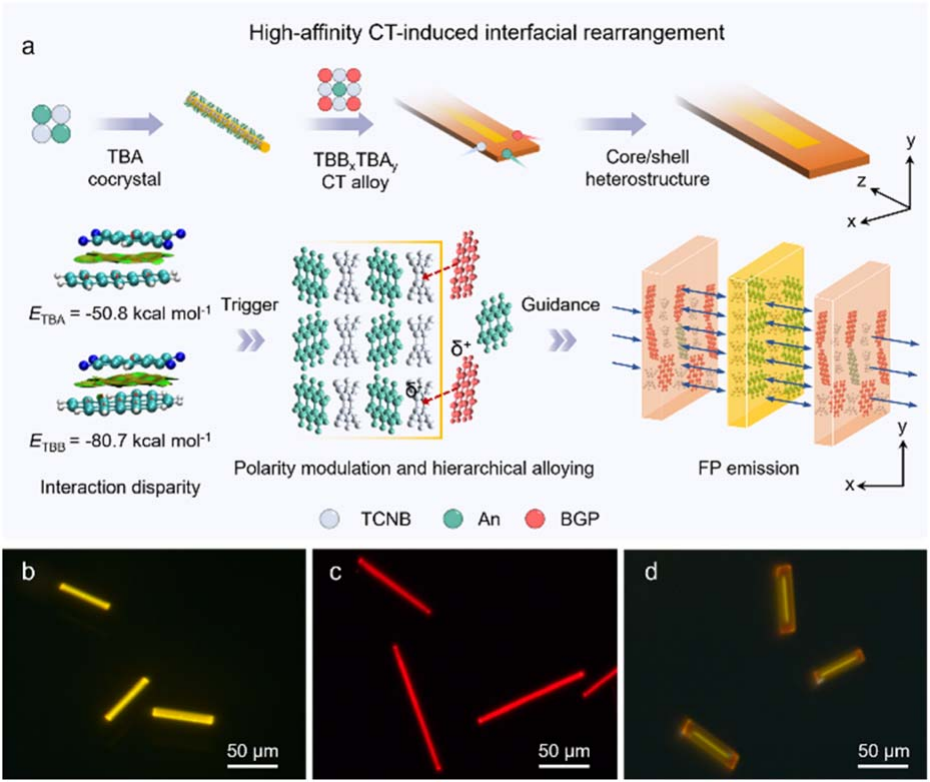
Description of directional self-assembly strategy for OCSHs. (a) Schematic illustration of interfacial polarity rearrangement-induced directional self-assembly proposed in this study. (b–d) FM images of (b) TBA, (c) TBB microrods and (d) organic core/shell heterostructures. Scale bars: 50 μm.

Herein, we proposed an interfacial polarity rearrangement-induced self-assembly strategy for constructing ordered OCSHs. By precisely modulating noncovalent interactions and interfacial polarity differences between distinct cocrystal systems, this approach enables directional and selective adsorption of components on the crystal surface, followed by layer-by-layer self-assembly (Fig. 1a). In a model system using 1,2,4,5-tetracyanobenzene-anthracene (TCNB-An: TBA) cocrystal as the core and TCNB-benzo[ghi]perylene_*x*_TBA_*y*_ (TBB_*x*_TBA_*y*_) alloy as the shell, the surface TCNB molecules of the core exhibit stronger CT interactions with BGP, inducing interfacial polarity rearrangement that drives the ordered growth of the shell layer. The resulting heterostructures feature aligned transition dipole moments and pronounced FP optical resonance behavior, enabling tunable optical logic outputs and well-defined energy transfer pathways. This strategy is applicable to a variety of organic cocrystal systems and enables dual-wavelength or white-light emission in selected structures, demonstrating excellent generality and scalability, which provides a new conceptual framework for the rational design of organic photonic devices.

## Results and discussion

The formation of the heterostructures fundamentally arises from differences in crystallization behavior and interfacial activity between the constituent crystals, necessitating individual investigation of the structural and electronic properties.^25–27^ The benzene ring skeleton of 1,2,4,5-tetracyanobenzene (TCNB) is highly planar and symmetric, with strong electron-withdrawing ability (Fig. S1a), allowing the rational design and controlled self-assembly of organic micro/nanomaterials with desirable optoelectronic properties. Anthracene (An) and benzo[ghi]perylene (BGP), prototypical electron-rich π-systems, served as donor components (Fig. S1b and c), and TCNB was shown to co-assemble controllably with the *via* strong CT interactions to form TCNB-An (TBA) or TCNB-BGP (TBB) cocrystals. TBA and TBB microrods, emitting yellow and red light, respectively, were synthesized *via* a facile One-dimensional (1D) self-assembly process in solution (Fig. 1b, c and S2), exhibiting photoluminescence (PL) peaks at 575 nm and 670 nm (Fig. S3), respectively. As shown in Fig. S4, the CT microrods show the emission colors with values of (0.51, 0.48) and (0.66, 0.34) according to CIE coordinate measurements. Scanning electron microscopy (SEM) images show that both TBA and TBB microrods possess well-defined 1D morphologies with distinct crystal planes, consistent with the simulated crystal models (Fig. S5–S7). Moreover, polar plots of polarized fluorescence display pronounced anisotropic emission for both cocrystals, indicating highly ordered molecular orientations (Fig. S8). X-ray diffraction (XRD) analysis further confirms the high crystallinity of both TBA and TBB cocrystals (Fig. S9). Notably, TBA and TBB crystals exhibit similar packing motifs, indicating a high degree of chemical and structural compatibility (Fig. S10 and S11).

During co-crystallization, donor–acceptor CT interactions induce the creation of novel hybridized orbitals.^28,29^ In the TBA and TBB cocrystals (−5.77 eV and −5.59 eV; Fig. S12 and S13), the highest occupied molecular orbitals (HOMOs) are primarily derived from An (−5.24 eV) and BGP (−5.53 eV), while the lowest unoccupied molecular orbital (LUMO) (TBA: −3.40 eV; TBB: −3.42 eV) originate from TCNB (−3.91 eV). The resulting energy gaps are markedly reduced relative to those of the individual components, indicating the formation of distinct CT states. Notably, TBB displays a narrower bandgap and a more pronounced red-shift in CT absorption and emission (Fig. S3). Due to the high compatibility between TBA and TBB in lattice parameters, molecular volumes, and energy level alignments (Fig. S14 and Table S1), spatially equivalent substitution can occur during the co-crystallization process, resulting in the formation of a stable TBB_*x*_TBA_*y*_ alloy phase. Based on the above discussion, a sequential self-assembly strategy was employed in this work, wherein TCNB and An were sequentially added dropwise onto the substrate, allowing for the preferential growth of regular TBA microrods. Subsequently, a solution containing TCNB and BGP was introduced. Due to the stronger interaction of TBB, interfacial complexation with TCNB molecules on the core-crystal surface was facilitated, inducing selective deposition and rearrangement of residual components on the core-crystal surface. Consequently, a TBB_*x*_TBA_*y*_ alloy shell was formed, ultimately constructing organic core/shell heterostructures (OCSHs) with the well-ordered structure and distinct interface (Fig. 1d).

**Fig. 2:**
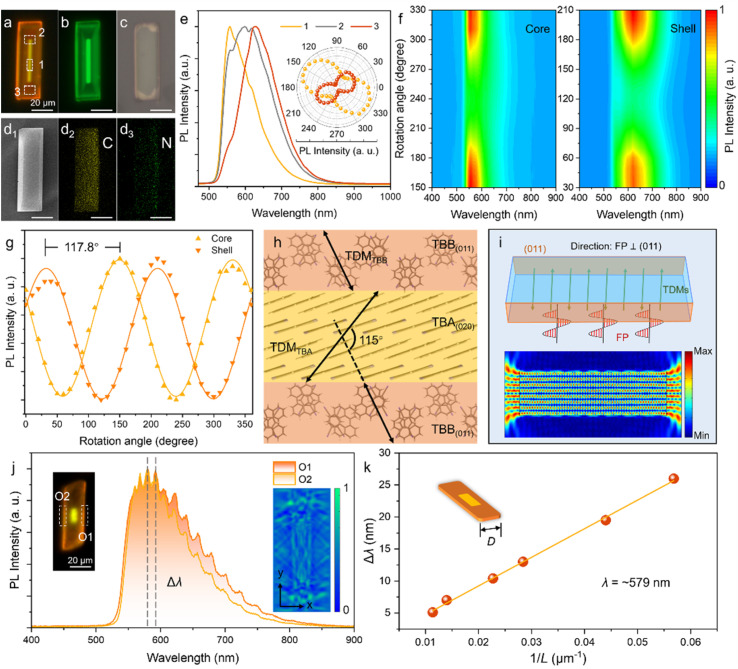
Optical and polarized emission characterization of OCSHs. (a–c) FM images of a single OCSH under (a) UV, (b) blue light, and (c) bright-field excitation, corresponding to 330–380 nm and 460–490 nm for (a) and (b). (d_1_) SEM image of the corresponding OCSH. (d_2_ and d_3_) The corresponding energy-dispersive X-ray spectroscopy (EDX) maps for C and N elements. (e) Micro-region PL (μ-PL) spectra obtained from various designated spots on the OCSH under *λ* = 395 nm excitation. Inset: related polar diagrams of the emission peak intensities. (f) Contour maps of polarization-resolved PL spectra obtained from the core and shell regions of heterostructure. (g) Polar plots of polarization-dependent emission intensities extracted from the core and shell regions. (h) The orientation of TDMs on the TBA (100) and TBB (011) crystal planes. (i) Top: schematic illustration of light paths in FP mode of OCSH. Bottom: simulated electric field distribution in the cavity. (j) μ-PL spectra collected along the long axis of the heterostructure. Top left: FM image of heterostructure showing the signal collection positions. Bottom right: simulated electric field propagation and standing wave patterns within the OCSH. (k) Linear relationship between the cavity length and the resonance peak spacing near 579 nm. All scale bars are 20 μm.

Fig. 2a shows the fluorescence microscopy (FM) image of a single core/shell heterostructure under UV excitation, exhibiting yellow emission at the center and orange emission at the edges. Under blue light illumination, both the core and shell layers exhibit varying intensities of green emission (Fig. 2b). Bright-field optical and SEM images confirm that the heterostructure maintains an overall continuous planar morphology without clear structural boundaries, indicating good compatibility between the constituent components (Fig. 2c and d_1_). The EDX mapping reveals that the carbon (C) and nitrogen (N) elements are uniformly distributed across the heterostructure, further confirming its continuous planar morphology without distinct structural boundaries (Fig. 2d_2_ and d_3_). According to Fig. 2e, emission at 560 nm corresponds to the core (position 1), whereas emission at 630 nm corresponds to the edge (position 3), consistent with the emission characteristics of pure TBA and TBB_*x*_TBA_*y*_ alloy crystals. Orbital analysis at the interface (Fig. S15) shows that the HOMO is primarily localized on BGP, while the LUMO resides on TCNB, indicating the formation of an interfacial CT state distinct from those in the core and shell. A new excited-state transition pathway may be introduced by the interfacial CT complex, resulting in an additional emission level and spectral peak (position 2, Fig. 2e).

To further investigate molecular orientation behavior in different regions of the core/shell heterostructure, polarization-dependent fluorescence spectra were collected separately from the core and shell areas, and contour maps were generated. As shown in Fig. 2e and f, emission intensity in both regions displays a pronounced angular dependence characterized by a distinct double-peak polarization pattern, indicating preferential molecular alignment along specific directions in both the cocrystal core and the alloy shell. The polarization emission angle corresponds to the directionality of photon emission during the radiative transition of excited-state molecules and is often correlated with the transition dipole moments (TDMs).^30,31^ Comparison of the extreme polarization angles reveals a difference of 117.8° (Fig. 2g), closely matching the angle between the TDM directions (Δ*θ* ≈ 115°) calculated *via* time-dependent density functional theory (TD-DFT) (Fig. 2h and S16). The molecular-scale ordered orientation directly influences the optical feedback characteristics of the heterostructure, playing a crucial role in the formation of Fabry–Pérot (FP) cavities.^32,33^ In the planar core/shell configuration, the directional transition between the cocrystal core and the alloy shell is achieved in an orderly manner at the molecular level. Specifically, the (020) crystal plane of TBA acts as the primary cavity region, while the (011) plane of TBB, as the main component of the shell layer, functions as an efficient reflective interface, as shown in Fig. 2h. Highly ordered planar FP cavities are synergistically formed together by these planes.

The optical path schematic shows that light in the heterostructure reflects repeatedly between two relatively flat end planes along the long axis, satisfying interference conditions and producing a stable standing-wave pattern (Fig. 2i). Simulated electric field distributions also exhibit periodic field intensity along the same axis, with alternating nodes and antinodes, characteristic of FP cavity resonance (*n* = 1.8). The interference condition is obeyed by this cavity mode:2*nL* = *mλ*where *n* is the effective refractive index, *L* is the cavity length (*L* = 2*D*, *D* representing the half-length), *m* is the integer mode number, and *λ* is the resonant wavelength. Fig. 2i further shows that the electric field decays rapidly along the short axis, indicating significant optical leakage and preventing multilayer reflection, thus interference fringes cannot form in this direction. It should be noted that whispering-gallery mode (WGM) requires total internal reflection around the cavity perimeter to establish circular standing-wave resonances, such feedback paths are absent here, ruling out WGM as the mechanism. Distinct FP interference fringes around 579 nm were observed in PL spectra collected along the long axis of the heterostructure, confirming the optical feedback capability of the cavity with a quality factor (*Q*-factor) of 115 (Fig. 2j). From the interference condition, the spacing between adjacent resonances (*m* and *m*+1) is given by:
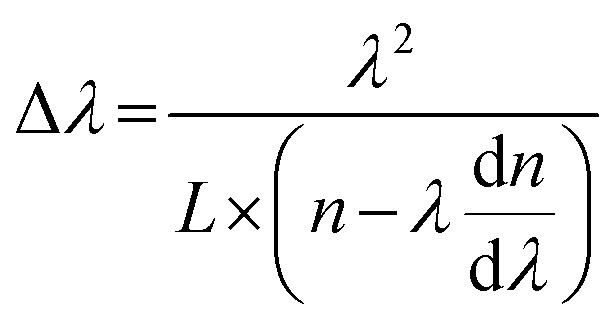
with d*n*/d*λ* accounting for material dispersion. A quantitative analysis of the cavity length (*L*) *versus* resonance spacing (Δ*λ*), as presented in Fig. 2k, reveals a linear relationship, which not only confirms the validity of the FP cavity model but also indicates that a high degree of internal order and interface quality within the heterostructure has been achieved.

**Fig. 3:**
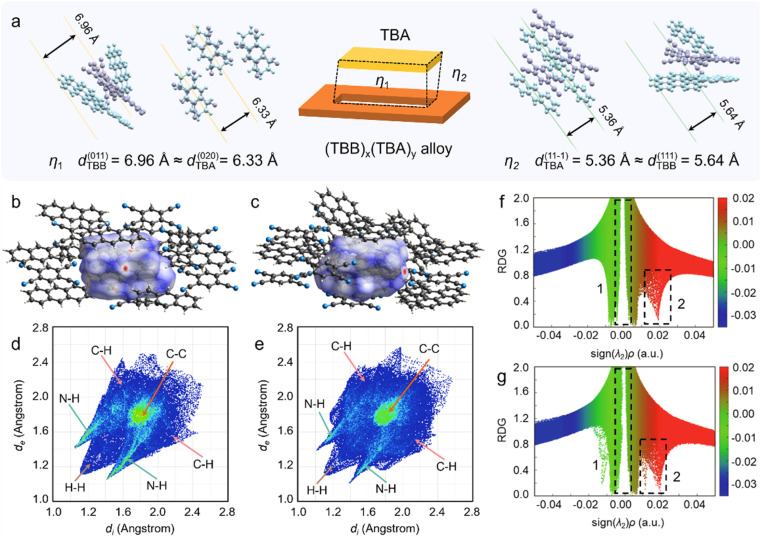
Crystal analysis and theoretical calculations. (a) Molecular packing model at the core/shell interface. (b and c) Hirshfeld surfaces of TBA and TBB, mapped with electron density. (d and e) 2D fingerprint plots of TBA and TBB. (f and g) RDG functions and Sign(*λ*_2_)*ρ* scatter plots of TBA and TBB.

To gain insight into the formation mechanism of core/shell heterostructures, we conducted a comprehensive investigation of interfacial packing behaviors and intermolecular interactions based on crystallographic analysis and theoretical calculations. TBA CT cocrystal adopts a monoclinic crystal system with unit cell parameters *a* = 9.441 Å, *b* = 12.650 Å, *c* = 7.299 Å, *α* = 90°, *β* = 93.11°, *γ* = 90°, whereas TBB CT cocrystal crystallizes in an orthorhombic system with parameters *a* = 9.6471 Å, *b* = 31.514 Å, *c* = 7.1332 Å, and *α* = *β* = *γ* = 90° (Table S1). As shown in Fig. S8, both TBA and TBB molecules adopt a face-to-face π–π stacking arrangement with homologous packing motifs, which facilitates the formation of TBB_*x*_TBA_*y*_ alloy phases and enables selective nucleation and epitaxial growth. In addition, the intrinsic lattice matching between the (020) plane of TBA microrods (*d* = 6.33 Å) and the (011) plane of TBB (*d* = 6.96 Å), as well as between the (11-1) plane of TBA and the (111) plane of TBB (*d*_TBA_ = 5.36 Å ≈ *d*_TBB_ = 5.64 Å) (Tables S2 and S3), promotes coherent interfacial growth (Fig. 3a). The calculated lattice mismatches (*η*) between TBA and TBB are 9.0% and 5.0%, respectively, values sufficiently low to support the formation of epitaxial OCSHs, which is further supported by XRD patterns of the core/shell samples (Fig. S9), which show characteristic diffraction peaks corresponding to both TBA and TBB cocrystals.

Further Hirshfeld Surface (HS) analysis reveals that TBA cocrystal exhibits more densely distributed red hotspot regions (Fig. 3b and c), indicating close intermolecular contacts where distances are shorter than the sum of the van der Waals radii. This indicates stronger intermolecular interactions at the TBA crystal surface.

Consistently, the corresponding 2D fingerprint plots show that N–H contacts account for 45.8% in TBA, significantly higher than in TBB (36.3%), highlighting the prevalence of polar interactions, particularly hydrogen bonding, in the TBA structure (Fig. 3d, e and S17). These interactions promote more ordered and directional molecular packing. The pronounced red regions on the HS further support the presence of stronger polar interactions, indicating that the TBA surface possesses higher polar activity, making it a favorable interfacial site for initiating subsequent self-assembly. Notably, the stronger CT interaction in TBB (|*E*_TBB_ = −80.7 kcal mol^−1^| > |*E*_TBA_ = −50.8 kcal mol^−1^|) facilitates the formation of a new interfacial CT state through electron donation from BGP to the TCNB molecules in the core layer (Fig. S15). This interfacial complexation, combined with polarity rearrangement, synergistically promotes the ordered alloying of the shell, revealing a molecular-level mechanism involving selective adsorption and charge-mediated coupling. Meanwhile, the proportions of C–H (15.7%) and H–H (27.8%) contacts in TBB are significantly higher than those in TBA (9.6% and 25.5%, Fig. S17–S19), indicating that TBB molecular packing relies more on weak, non-directional van der Waals interactions. This relatively loose and adjustable packing mode endows TBB with greater interfacial adaptability, allowing it to flexibly conform to and integrate with the TBA crystal surfaces under polarity-driven perturbation. Such adaptability further facilitates the integration of heterogeneous shell components and the construction of structurally continuous heterostructures.

To further elucidate the differences in intermolecular noncovalent interactions between the two CT cocrystals, we analyzed scatter plots of the reduced density gradient (RDG) *versus* the Sign(*λ*_2_)*ρ* function (Fig. 3f and g). Compared to TBA, the TBB cocrystal exhibits more prominent regions of weak interactions (green) at higher electron densities, along with broader zones of steric hindrance (brown). These features effectively restrict intermolecular vibrations and enhance molecular rigidity, while also suppressing the loss of excitation energy *via* nonradiative vibrational relaxation.^34^ The unique combination of high-density and high-rigidity interactions facilitates the formation of a well-confined shell layer with favorable optical properties, providing a structural foundation for efficient coupling between directional TDMs and FP microcavity electric field. Collectively, these data further validate that the interfacial polarity-induced mechanism driven by CT interactions enables heterogeneous integration between the core layer and the shell layer of the multicomponent alloy, resulting in the formation of structurally continuous and interfacially well-defined organic core/shell heterostructures.

Owing to the highly controllable molecular assembly and versatile tunability of electronic states, organic heterostructures have emerged as key material systems for constructing micro/nanoscale photonic functional devices.^35,36^ In particular, excited-state transport is governed by the stratified core/shell interface in heterostructures, and both light propagation direction and emission behavior are strongly affected.^37,38^

**Fig. 4:**
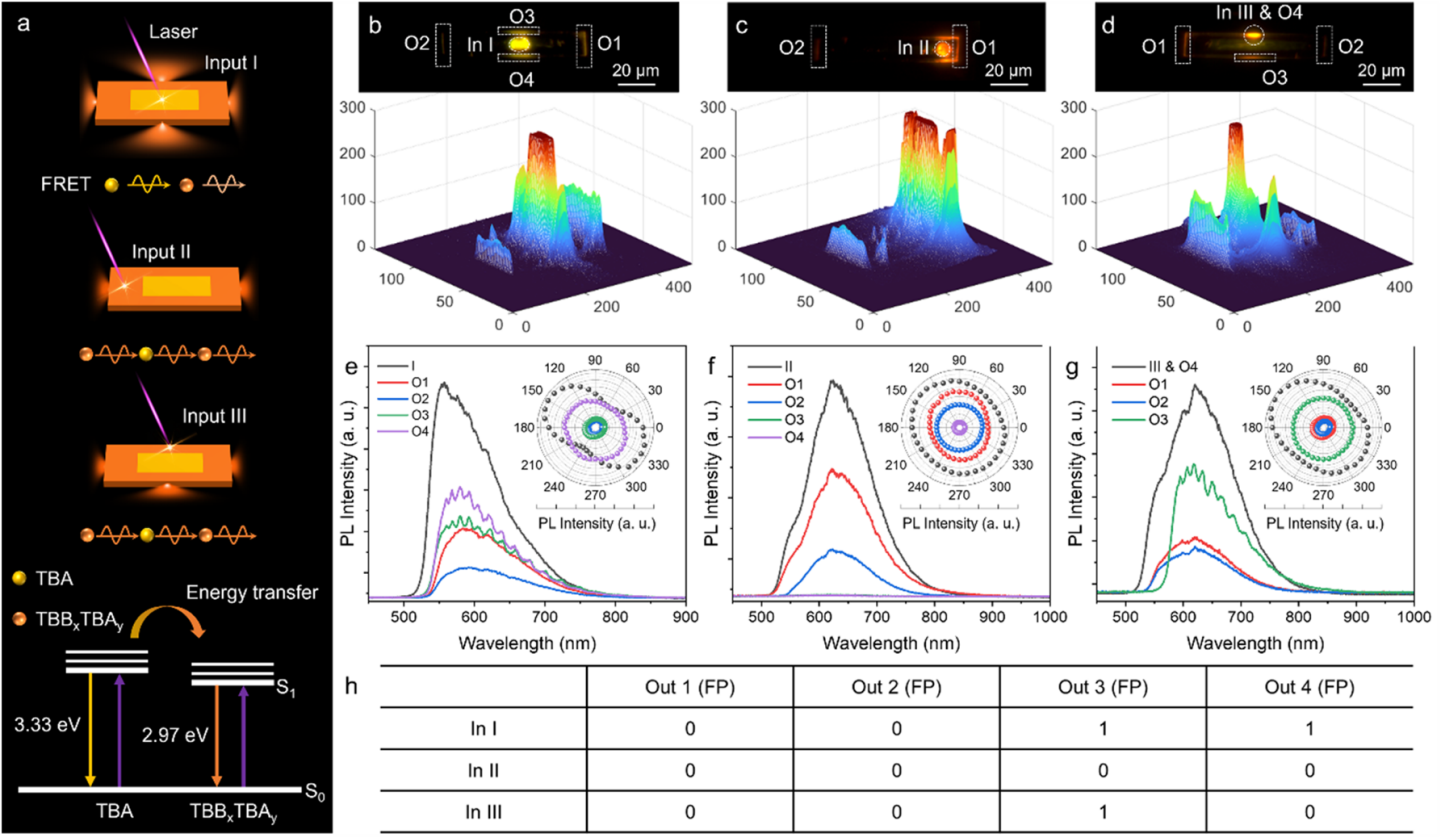
Optical logic response in core/shell heterostructures. (a) Schematic illustration of light propagation and ET processes within OCSHs. (b–d) FM images of heterostructure under focused laser excitation at different positions, with corresponding spatial intensity maps of emitted light. Scale bars: 20 μm. (e–g) Spatially resolved PL spectra were recorded from both excitation and output regions under different excitation sites. Insets: corresponding polar plots of peak emission intensities. (h) Summary table of the optical logic responses exhibited by OCSHs.

To investigate photonic transport behavior, we used a custom-built optical microscope to measure the waveguiding properties of TBA and TBB cocrystals (Fig. S20). Specifically, through precisely moving the excitation laser spot (*λ* = 395 nm) along individual organic microrods, bright yellow and red emissions were observed at the tips of the TBA and TBB microstructures (Fig. S21a and S22a), respectively. Distance-dependent PL spectra collected from the tips showed a gradual decrease in PL intensity with increasing photon propagation distance (Fig. S21b and S22b). By fitting the PL intensity decay using a single-exponential function: *I*_tip_/*I*_body_ = *A* exp(–*R*D), the optical loss coefficients (*R*) were determined. The optical loss coefficients of the TBA and TBB microrods were determined to be 0.012 dB μm^−1^ and 0.04 dB μm^−1^ (Fig. S21c and S22c), respectively, indicating excellent waveguiding capabilities and long-range energy transport performance. As shown in Fig. 4a, TBA enables an energetically favorable energy transfer (ET) from TBA to TBB due to the larger energy gap. Moreover, the significant spectral overlap between the absorption of TBB and the emission of TBA further supports the occurrence of Förster resonance energy transfer (FRET) (Fig. S3).

As shown in Fig. 4b–d, we systematically investigated the light transport behavior and emission characteristics of OCSHs under various excitation positions. The presence or absence of distinct FP interference fringes in the output spectra served as an optical encoding criterion: a clear FP signal was assigned a value of “1”, whereas the absence of interference was designated as “0” (Fig. 4h). When the excitation was applied to the yellow-emissive TBA cocrystal core (In I), the emitted photons were efficiently absorbed by the outer TBB_*x*_TBA_*y*_ alloy shell, leading to energy conversion and orange emission (Fig. 4b). As a result, all PL spectra collected at the edges (out 1–4) displayed identical emission peaks (Fig. 4e). Notably, FP interference fringes were observed only at outputs along the longitudinal axis of the heterostructure, corresponding to a logic output of “0011”. When the excitation was applied to different regions of the shell (In II and In III) (Fig. 4c and d), the low-energy photons from the TBB_*x*_TBA_*y*_ shell were unable to excite the higher-energy states of the TBA core, resulting in passive photon propagation through the structure. Similarly, FP interference signals appeared only at the outputs along the long axis (Fig. 4f and g), corresponding to logic codes of “0000” and “0010”, respectively. Above all, direction-dependent light propagation and conversion capabilities are exhibited by OCSHs, and multi-state logic encoding based on FP interference signals is supported, enabling programmable control of photon transport under different excitation conditions.

**Fig. 5:**
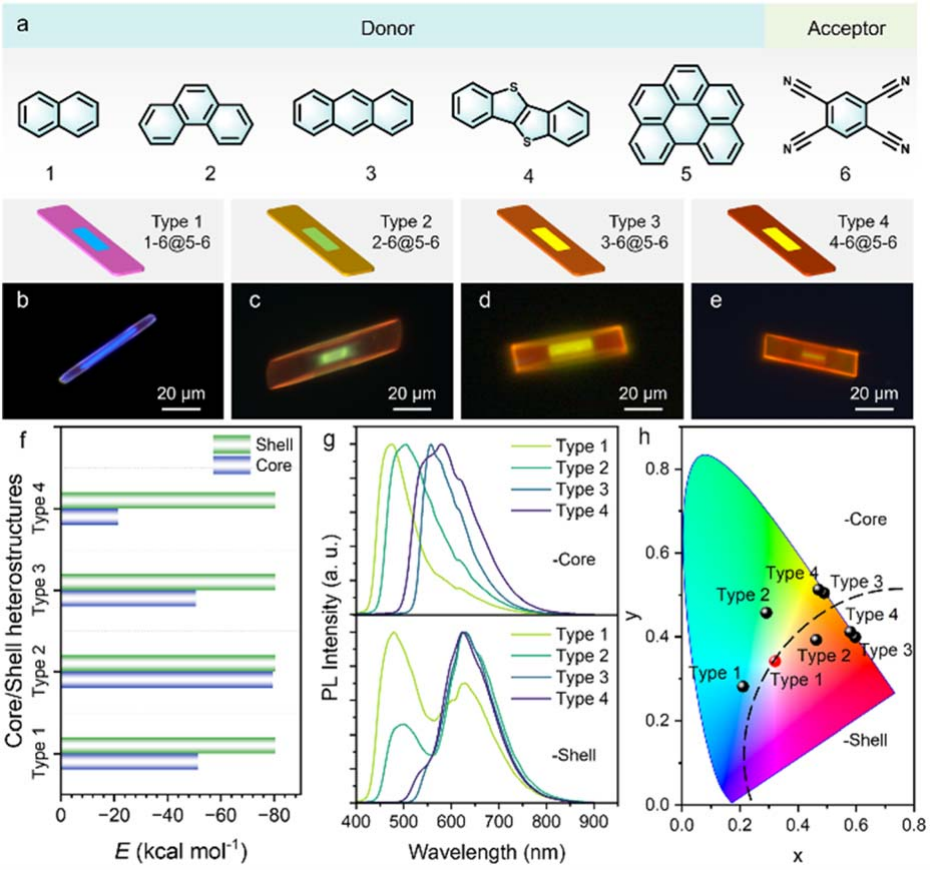
Universality of interfacial polarity-driven self-assembly strategy. (a) Depicted molecular structures of the donor (1–5) and acceptor (6) species. (b–e) FM images of OCSHs constructed from different combinations of organic CT cocrystals. (f) Comparison of the noncovalent interaction strengths between the core and shell cocrystals in various OCSHs. Scale bars: 20 μm. (g) Spatially resolved μ-PL spectra collected from the core and shell regions of various heterostructures. (h) CIE chromaticity diagrams corresponding to the emission positions shown in (g).

In order to verify the universality of the surface polarity rearrangement-induced self-assembly mechanism, we selected several representative electron donor–acceptor pairs (Fig. 5a and S23) to construct a series of OCSHs featuring well-defined core/shell domains (Fig. 5b–e). The shell layers are composed of alloys formed from different molecular components in all systems. Comparative analysis of intermolecular noncovalent interaction strengths (Fig. 5f) revealed that molecular interactions within the shell domain are generally stronger than those in the core.

This disparity in interaction strength facilitates the preferential growth of the shell on the core cocrystal surface, promoting directional molecular adsorption and enabling the formation of heterostructures with clear interfaces and well-ordered architectures. Spatially resolved PL spectra (Fig. 5g) and the corresponding CIE chromaticity diagrams (Fig. 5h) further elucidate the emission modulation characteristics of these heterostructures. Notably, certain alloy shell systems exhibit dual-wavelength emissions and white-light features in the CIE diagram (Fig. 5h), indicating that multiple emissive centers can cooperatively radiate through molecular combination tuning. These findings not only demonstrate the generality and effectiveness of the surface polarity rearrangement-induced self-assembly strategy but also highlight its potential for designing multicolor and white-light-emitting devices.

## Conclusions

In summary, this study systematically proposed and validated an interface polarity rearrangement-driven strategy for constructing OCSHs. By precisely regulating intermolecular noncovalent interactions, crystallization behavior, and interfacial packing modes, we achieved controllable assembly and functional integration of core/shell heterostructures. The ordered molecular alignment significantly enhances coupling with the FP cavity standing wave field, thereby boosting interference resonance and directional emission properties, and ultimately enabling efficient optical waveguiding. This generalizable strategy offers a new avenue for structure-function co-regulation and holds great promise for extension to a broader range of donor–acceptor systems, with potential applications in organic transistors, optical logic devices, and quantum photonics.

## Author contributions

J. Feng led the study by designing the methods, conducting the experiments, analyzing the data, and drafting the manuscript, including preparing the figures. Z.-Y. Geng, Y. Zong, C.-Z. Wang and S.-H. Chen contributed to organizing the SI. H.-T. Lin, L.-W. Xie and X.-D. Wang provided funding and oversaw the overall direction of the project. All authors reviewed and provided feedback on the manuscript.

## Conflicts of interest

There are no conflicts to declare.

## Supplementary Material

SC-016-D5SC05873B-s001

## Data Availability

The data supporting this article can be found in the SI. Supplementary information: materials used; preparation details; test analysis method; FM images, PL spectra and CIE chromaticity diagram, simulated growth morphology, and theoretical calculation of the crystals; FM images of supplementary crystals; crystallographic data of the crystals. See DOI: https://doi.org/10.1039/d5sc05873b.
