# Supplementary material for: Interfacial polarity-driven self-assembly of organic core/shell heterostructures with directional Fabry–Pérot resonance
Source: Chem Sci. 2025 Sep 24;16(42):19769–76. doi: 10.1039/d5sc05873b (PMC12459620; doi:10.1039/d5sc05873b)
Supplement: SC-016-D5SC05873B-s001 [file SC-016-D5SC05873B-s001.pdf]

Supplementary Information (SI) for Chemical Science.
This journal is © The Royal Society of Chemistry 2025

Supporting Information

Interfacial Polarity-Driven Self-Assembly of Organic Core/Shell Heterostructures with Directional Fabry-Pérot Resonance

Jin Feng,[a] Zhen-Yu Geng,[a] Yi Zong,[b] Chuan-Zeng Wang,[a] Shu-Hai Chen,[a] Hong-Tao Lin[a], Li-Wei Xie*[c] and Xue-Dong Wang*[b]*

[a] School of Chemistry and Chemical Engineering, Shandong University of Technology, Zibo 255000, China;

[b] Institute of Functional Nano & Soft Materials (FUNSOM), Jiangsu Key Laboratory for Carbon-Based Functional Materials & Devices, Soochow University, Suzhou 215123, China;

[c] Suzhou Key Laboratory for Radiation Oncology, Department of Radiotherapy and Oncology, The Second Affiliated Hospital of Soochow University, Suzhou 215004, China.

E-mail: linht@sdut.edu.cn (H.-T. Lin); wangxuedong@suda.edu.cn (X.-D. Wang); xlw511605617@163.com (L.-W. Xie)

Table of Contents

1. Experimental Details 4
1.1 Materials 4
1.2 Preparation of one-dimensional (1D) microrods 4
1.3 Preparation of Organic Core/Shell Heterostructures 4
1.4 Theoretical Calculations of Crystal Structures 5
1.5 Characterization of Crystal Structure 5
2. Results and Discussion 6
Figure S1. The electrostatic potential maps of molecules 6
Figure S2. The molecular stacking modes and FM images of cocrystals 6
Figure S3. The absorption spectra and PL spectra 7
Figure S4. CIE chromaticity diagram of cocrystals 7
Figure S5. SEM images of microrods 8
Figure S6. The predicted growth morphology of cocrystals 8
Figure S7. The simulated equilibrium morphology of cocrystals 8
Figure S8. Polar coordinate images 9
Figure S9. XRD patterns of cocrystals and heterostructures 9
Figure S10. Unit cell structure of TBA cocrystals 10
Figure S11. Unit cell structure of TBB cocrystals 10
Figure S12. Molecular orbital diagrams of An, TCNB and TBA 11
Figure S13. Molecular orbital diagrams of BGP, TCNB and TBB 11
Figure S14. The VDW radius of molecules 12
Figure S15. Molecular orbital diagrams of BGP-TCNB-An interface state 12
Figure S16. The electric TDMs of TBA and TBB 13
Figure S17. The proportion of interactions in TBA and TBB 13
Figure S18. 2D fingerprint plots of TBA 14
Figure S19. 2D fingerprint plots of TBB 14
Figure S20. Schematic diagram of the optical characterization 15
Figure S21. Position-dependent PL response of TBA 15
Figure S22. Position-dependent PL response of TBB 16
Figure S23. PL spectra, FM images and CIE diagram of CT cocrystals 16
Table S1. Crystal data and structure refinement for TBA and TBB 17
Table S2. Attachment energies of TBA crystal facets (*hkl*) 18

Table S3. Attachment energies of TBB crystal facets (*hkl*) 19

3. References 20

1. Experimental Details

1.1 Materials

1,2,4,5-Tetracyanobenzene (TCNB, CAS: 712-74-3), Anthracene (An, CAS: 120-12-7), Benzo[ghi]perylene (BGP, CAS: 191-24-2), Naphthalene (Na, CAS: 91-20-3), Phenanthrene (Phen, CAS: 85-01-8), Benzo[b]benzo[4,5]thieno[2,3-d]thiophene (BBTT, CAS: 248-70-4) were purchased from Innochem. Dichloromethane (DCM, analysis grade) and ethanol (EtOH, analysis grade) were purchased from Beijing Chemical Agent Ltd., China. All chemicals were used directly without additional purification.

1.2 Preparation of one-dimensional (1D) microrods

TCNB-based cocrystals were prepared via a simple solvent evaporation method.[1,2] Specifically, 1.78 mg of TCNB and 1.78 mg of An were dissolved in 1 mL of DCM to prepare a stock solution of TCNB-An (TBA). This stock solution was then mixed with 2 mL of EtOH and stirred thoroughly. The resulting mixture was drop-cast onto a quartz substrate and allowed to evaporate slowly at room temperature, resulting in the formation of yellow TBA microrods. Similarly, 1.78 mg of TCNB and 2.76 mg of BGP were dissolved in 1 mL of DCM to prepare a TCNB-BGP (TBB) stock solution, which was subsequently mixed with 2 mL of EtOH and stirred thoroughly. After drop-casting the mixture onto a quartz substrate and allowing the solvent to evaporate slowly, red TBB microrods were obtained. The same procedure can be applied to prepare 1D cocrystal microrods of TCNB with Na, Phen or BBTT, using the corresponding stoichiometric ratios.

1.3 Preparation of Organic Core/Shell Heterostructures

Specifically, 1.78 mg of TCNB and 1.78 mg of An were dissolved in 2 mL of DCM to prepare a TBA stock solution, which was subsequently mixed with 2 mL of EtOH to obtain a homogeneous TBA solution. Simultaneously, 0.89 mg of TCNB and 1.38 mg of BGP were dissolved in 4 mL of DCM to prepare a TBB stock solution, which was then mixed with 4 mL of EtOH to yield the corresponding homogeneous solution. Subsequently, 200 μL of the TBA solution was drop-cast onto a quartz substrate and immediately covered with a glass Petri dish to control the solvent evaporation rate. After 30 seconds, 200 μL of the TBB solution was added to the same substrate and again immediately covered with a glass dish. Upon complete solvent evaporation, organic core/shell heterostructures ware successfully obtained.

1.4 Theoretical Calculations of Crystal Structures

The crystal growth morphologies of the cocrystals were simulated using Materials Studio. The molecular packing modes of the crystal structures were visualized using Mercury. Hirshfeld surface analysis and 2D fingerprint plots were performed with Crystal Explorer.[3] All energy-level calculations were carried out using Gaussian 16, employing the B3LYP functional with the 6-311G(d) basis set.[4] Van der Waals (VDW) radii and reduced density gradient (RDG) functions were computed with the Multiwfn program. Time-dependent density functional theory (TDDFT) calculations were conducted using the PBE0 functional with Grimme's dispersion correction and the 6-31G(d) basis set to optimize the transition dipole moments (TDMs) of the cocrystals.[5] The electric field distribution of the Fabry-Pérot (FP) cavity was simulated using MATLAB and COMSOL.

1.5 Characterization of Crystal Structure

The morphology and size of the microstructures were observed using field-emission scanning electron microscopy (FESEM, Carl Zeiss, Supra 55, Germany). X-ray diffraction (XRD) patterns were recorded using a D/max 2400 diffractometer equipped with a Cu Kα radiation source (λ = 1.54050 Å). The scanning range was 2θ = 5° to 35°, and the samples were fixed on quartz substrates during the measurements. Bright-field and fluorescence microscopy (FM) images were acquired using an upright fluorescence microscope (Leica DM4000 M, Germany) equipped with a mercury lamp excitation source and a UV bandpass filter (330-380 nm). The photoluminescence (PL) spectra of the organic microstructures were measured using a custom-built micro-region PL (μ-PL) system. A 395 nm laser was focused onto the sample through a Nikon CFLU Plan 100× objective (numerical aperture N.A. = 0.8) to excite specific regions, generating a laser spot with a diameter of approximately 2 μm. The emitted PL signals were collected and coupled into a grating spectrometer (Princeton Instruments ARCSP-2356) and recorded by a thermoelectrically cooled CCD detector (Princeton Instruments PIX-256E). Notably, this CCD detector possesses spatial resolution (~1 μm) to minimize crosstalk between different emission signals. PL micrographs were captured using an inverted fluorescence microscope.

2. Results and Discussion

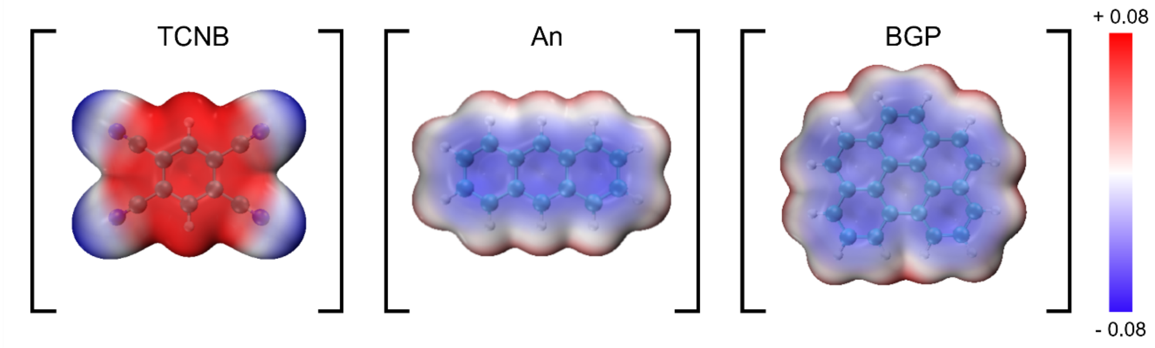

Figure S1. The electrostatic potential maps of TCNB, An, and BGP molecules.

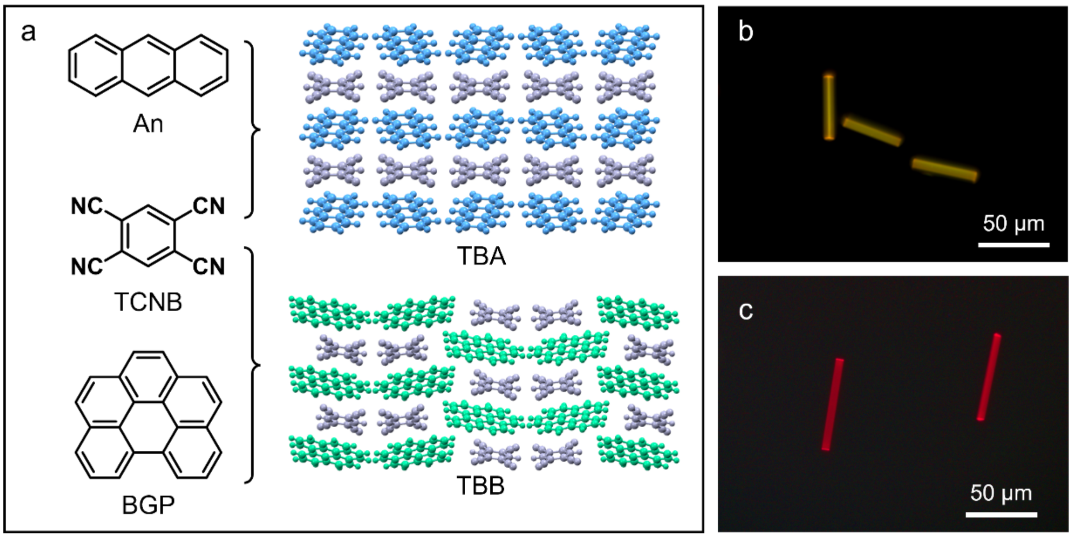

Figure S2. (a) The molecular stacking modes of An, TCNB, and BGP molecules in the self-assembled TBA and TBB cocrystals. (b-c) FM images of (b) TBA and (c) TBB cocrystals. Scale bars: 50 μm.

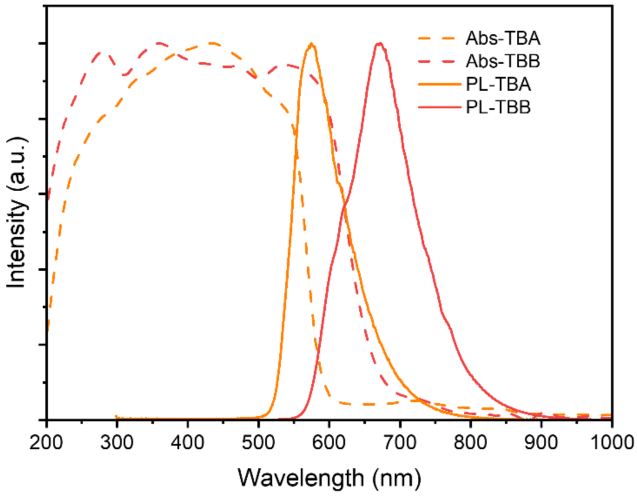

Figure S3. Spatially resolved absorption spectra and PL spectra of TBA (orange lines) and TBB (red lines) cocrystals.

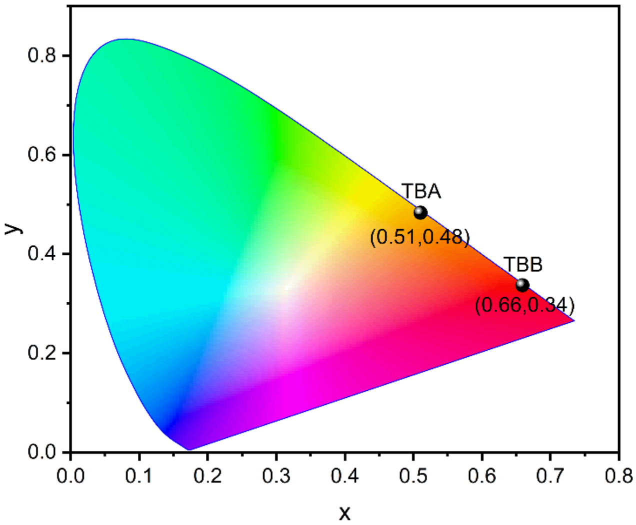

Figure S4. CIE chromaticity diagram corresponding to TBA and TBB.

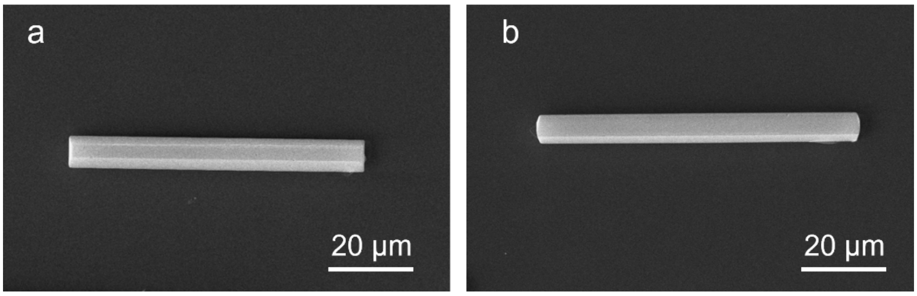

Figure S5. SEM images of (a) TBA and (b) TBB microrods. Scale bars: 20 µm.

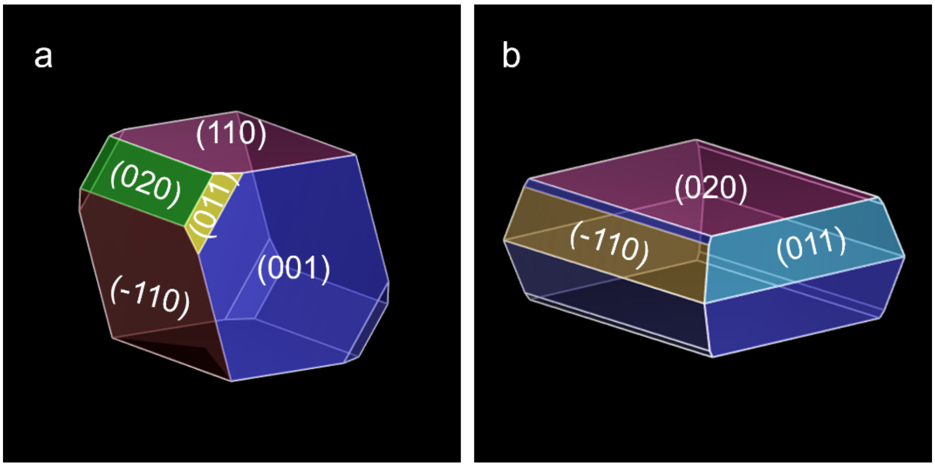

Figure S6. (a) The predicted growth morphology of TBA cocrystal. (b) The predicted growth morphology of TBB cocrystal.

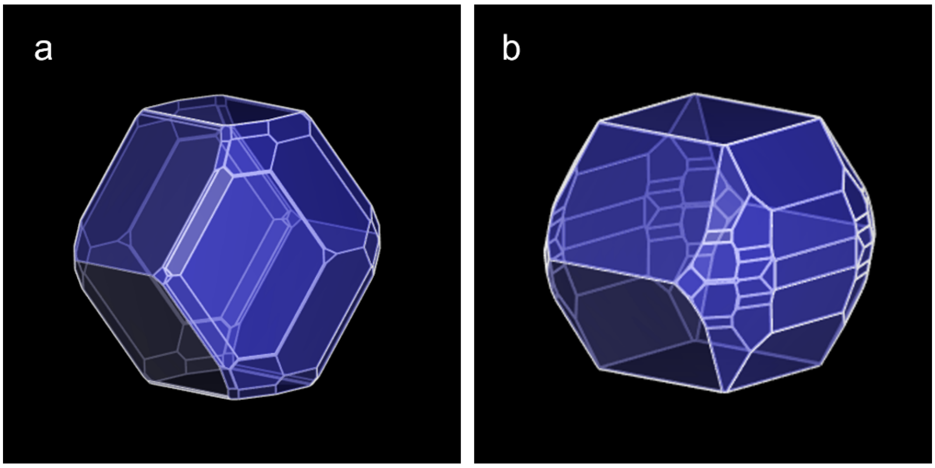

Figure S7. (a) The simulated equilibrium morphology of TBA cocrystal. (b) The simulated equilibrium morphology of TBB cocrystal.

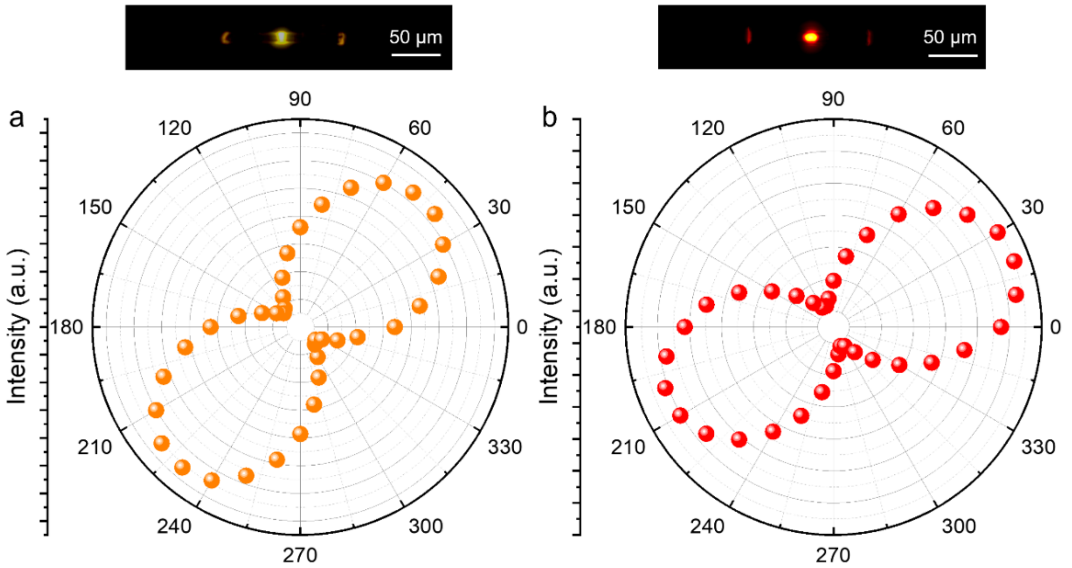

Figure S8. (a) FM image of the single TBA microrod under laser excitation (λ = 395 nm) and polar coordinate images at different peak intensities, scale bar: 50 μm. (b) FM image of the single TBB microrod under laser excitation (λ = 395 nm) and polar coordinate images at different peak intensities, scale bar: 50 μm.

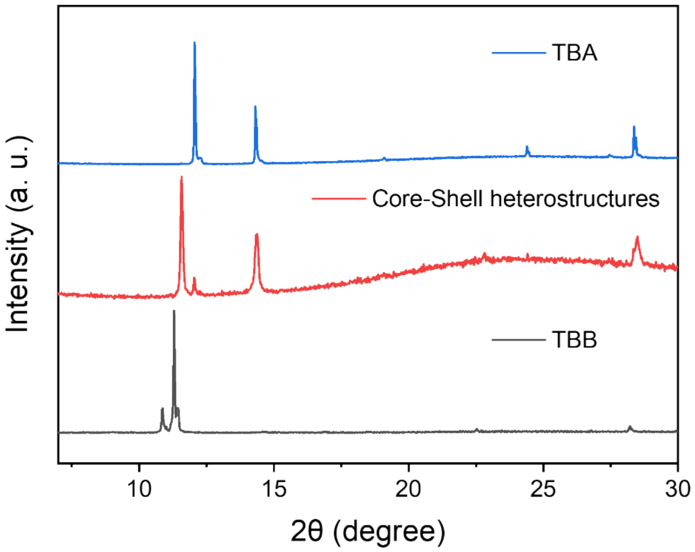

Figure S9. XRD patterns of the TBA, TBB and Core-Shell heterostructures.

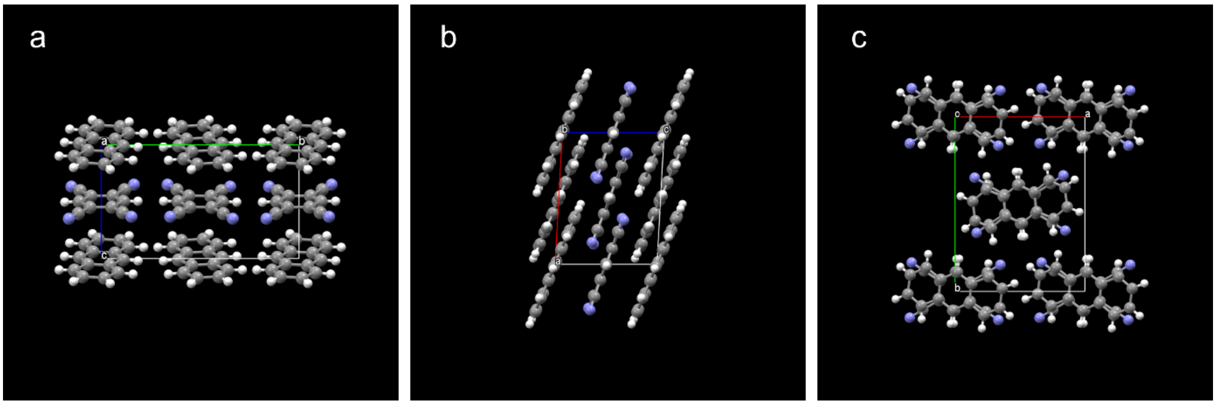

Figure S10. Unit cell structure of TBA cocrystals. Singe crystal packing of TBA view along a direction (a), view along b direction (b) and view along c direction (c).

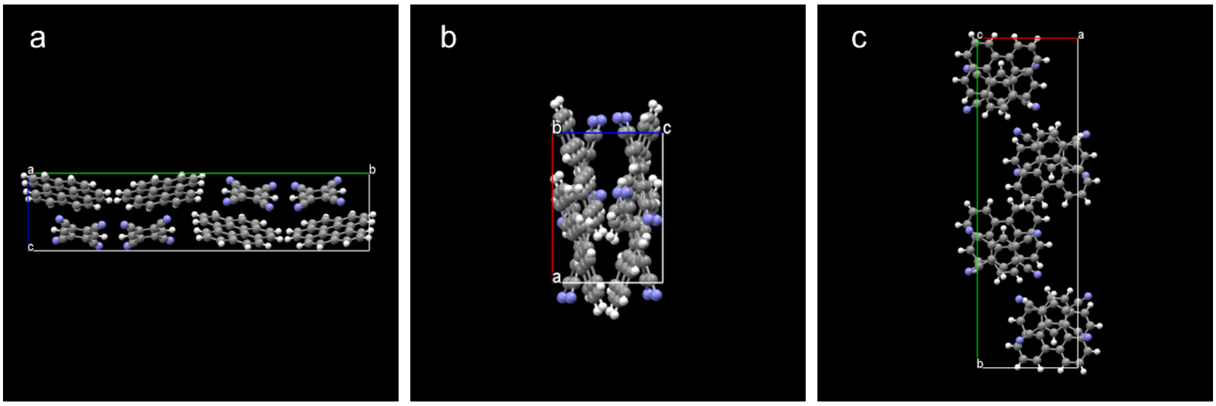

Figure S11. Unit cell structure of TBB cocrystals. Singe crystal packing of TBB view along a direction (a), view along b direction (b) and view along c direction (c).

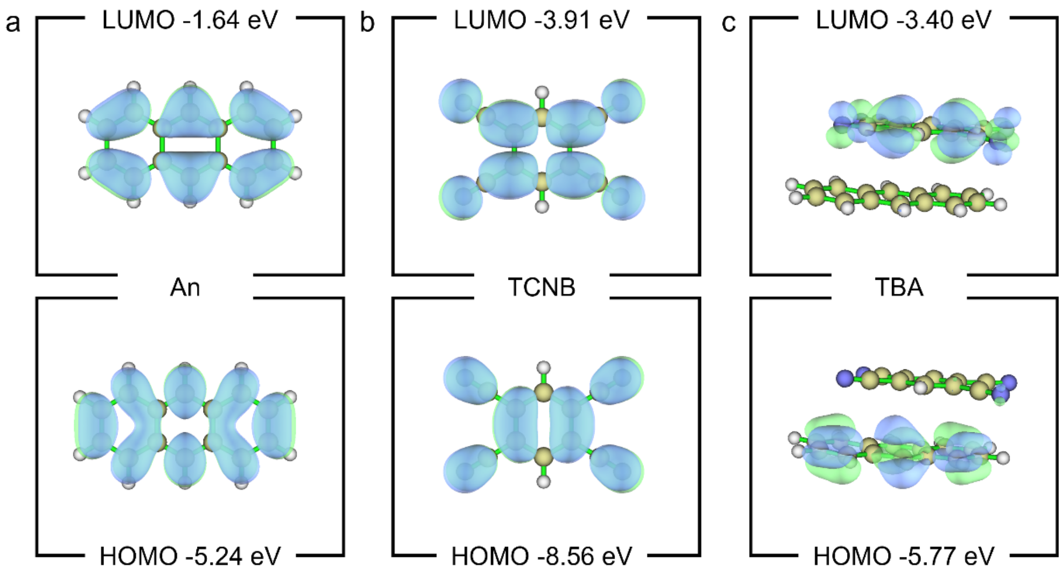

Figure S12. Molecular orbital diagrams of An, TCNB and TBA cocrystal obtained by DFT calculations using Multiwfn.

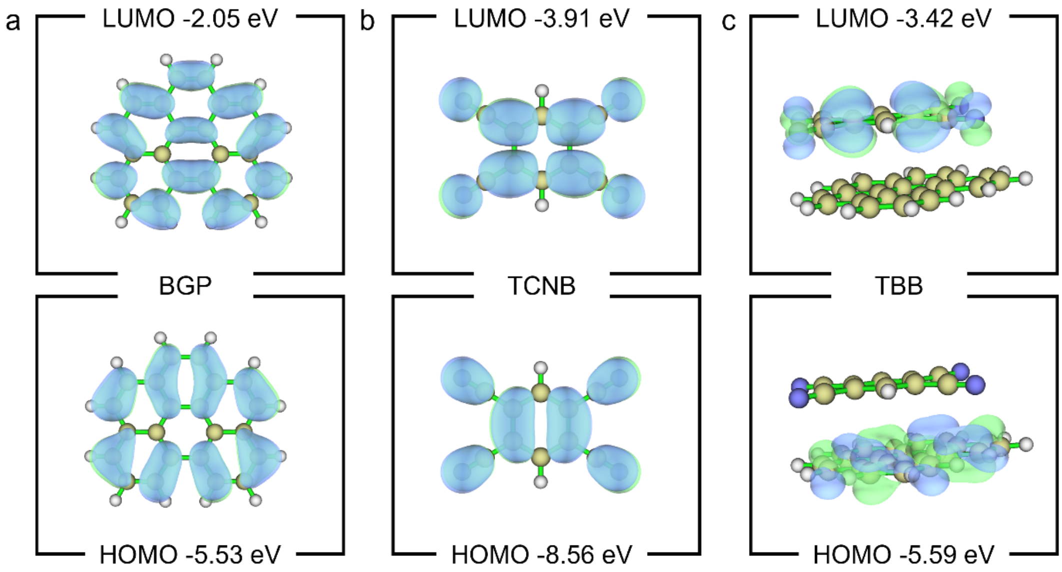

Figure S13. Molecular orbital diagrams of BGP, TCNB and TBB cocrystal obtained by DFT calculations using Multiwfn.

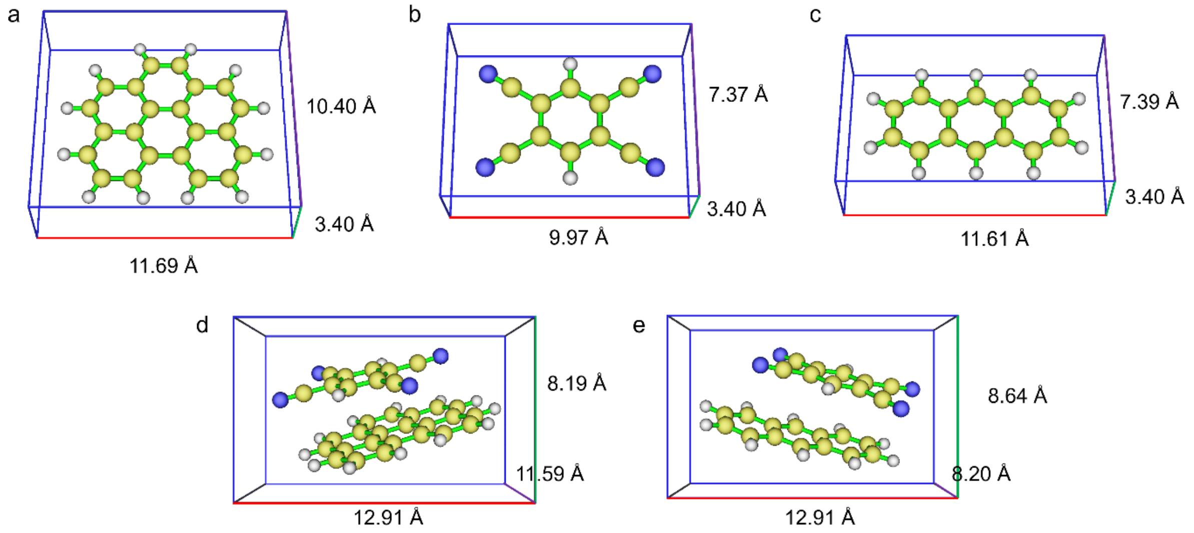

Figure S14. (a-e) The van der Waals (VDW) radius of (a) BGP, (b) TCNB, (c) An, (d) TBB and (e) TBA are determined using Multiwfn, where the VDW surface is defined based on the lengths of the three sides of the cube.

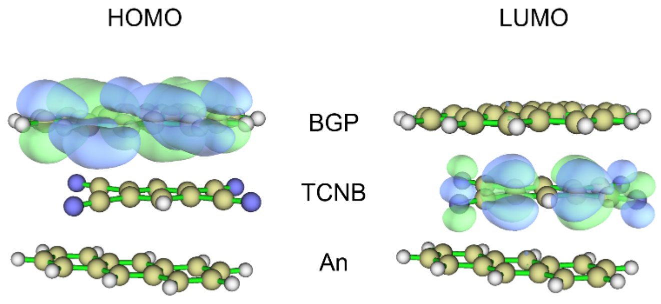

Figure S15. Molecular orbital diagrams of BGP-TCNB-An interface state obtained by DFT calculations using Multiwfn.

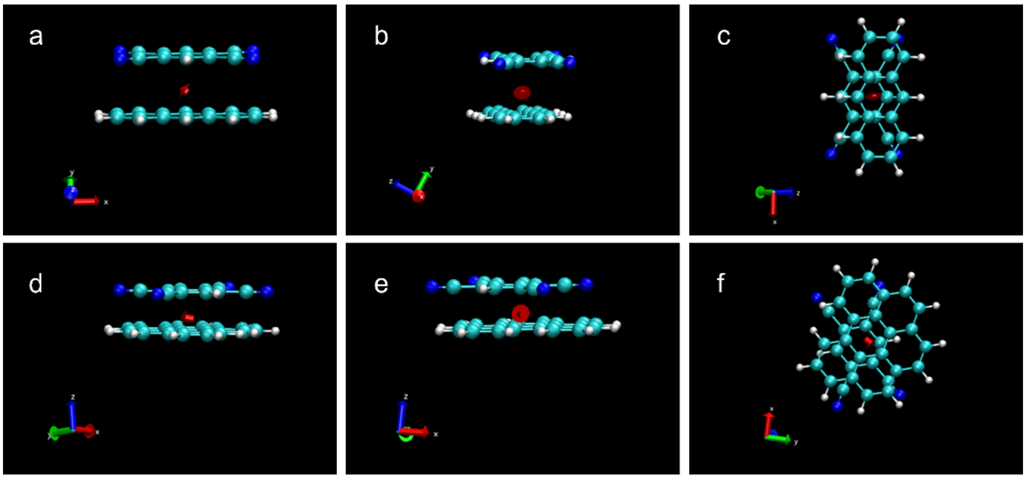

Figure S16. Simulated visualizations of the electric transition dipole moments (TDMs) vector components of (a-c) TBA and (d-f) TBB from different viewing angles.

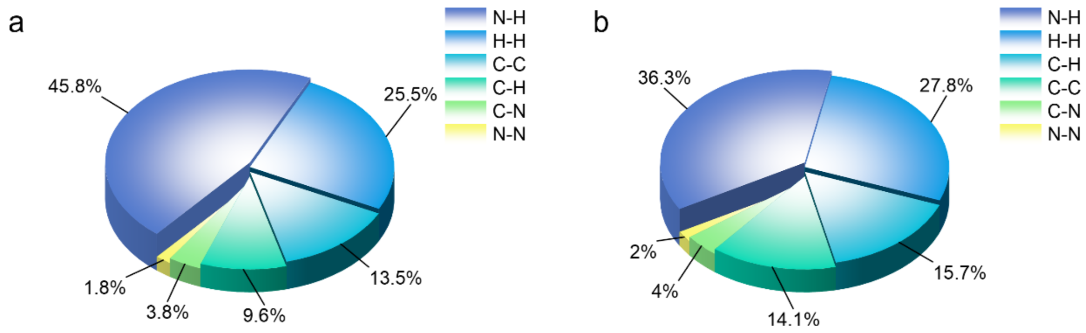

Figure S17. The proportion of different type of intermolecular interactions in (a) TBA and (b) TBB.

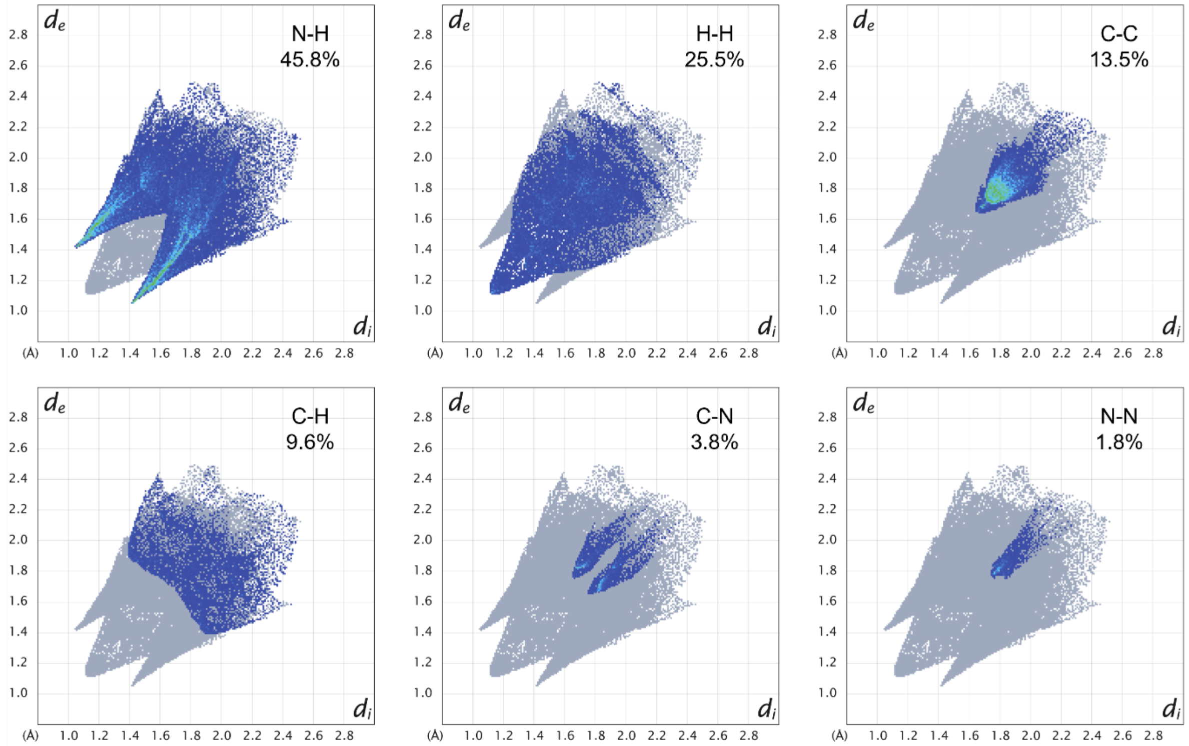

Figure S18. 2D fingerprint plots of different types of intermolecular interactions in the TBA cocrystal.

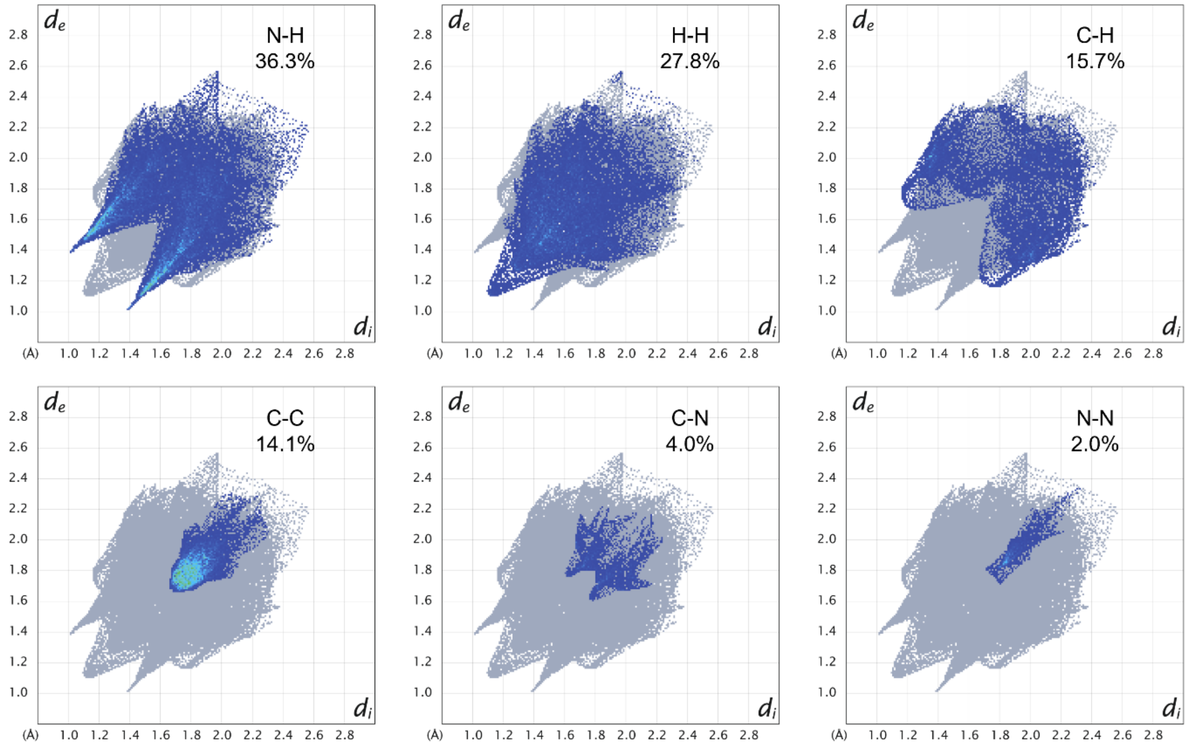

Figure S19. 2D fingerprint plots of different types of intermolecular interactions in the TBB cocrystal.

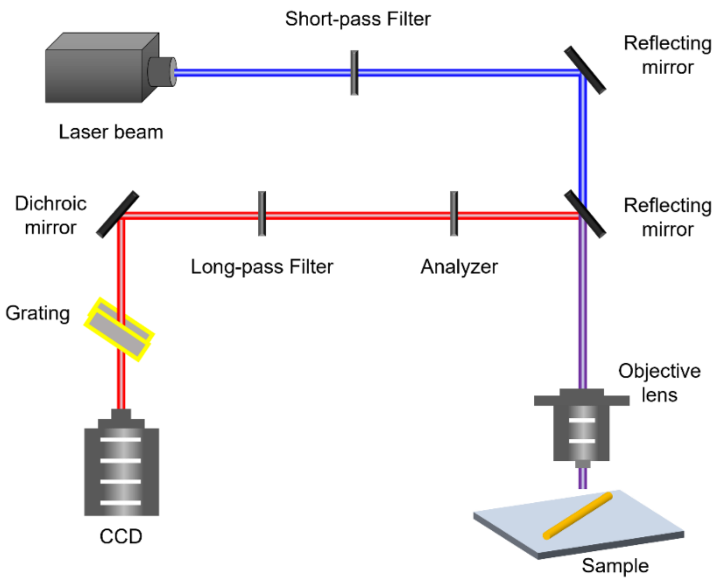

Figure S20. Schematic diagram of the experimental device for the optical characterization. The PL spectra of the organic microcrystals were measured by locally exciting the samples with a 395 nm laser using a focused beam, which had a diameter of approximately 2 μm and was directed through an objective lens (Nikon CFLU Plan, 50×, N.A. = 0.8). The power at the input was altered by the neutral density filters.

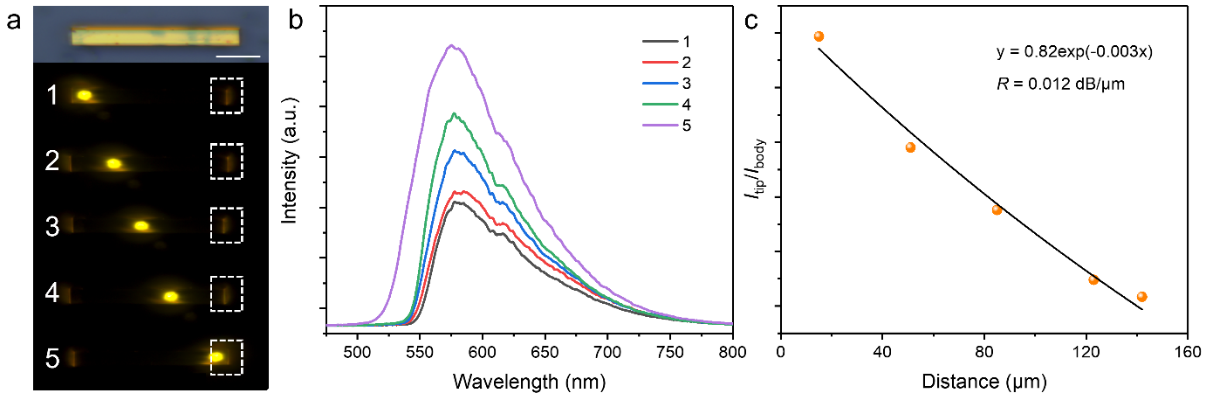

Figure S21. (a) FM images of a single TBA microrod excited at different positions using a laser beam (λ = 395 nm). Scale bar: 50 μm. (b) Spatially resolved photoluminescence spectra corresponding to different propagation distances in (a). (c) Variation of the intensity ratio between the tip (I_{tip}) and the body (I_{body}) as a function of distance (d) in (a).

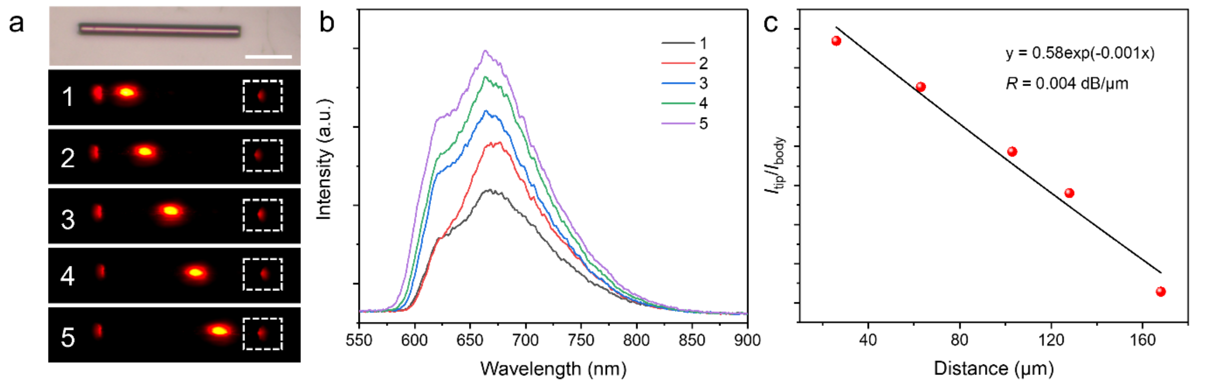

Figure S22. (a) FM images of a single TBB microrod excited at different positions using a laser beam (λ = 395 nm). Scale bar: 50 μm. (b) Spatially resolved photoluminescence spectra corresponding to different propagation distances in (a). (c) Variation of the intensity ratio between the tip (I_{tip}) and the body (I_{body}) as a function of distance (d) in (a).

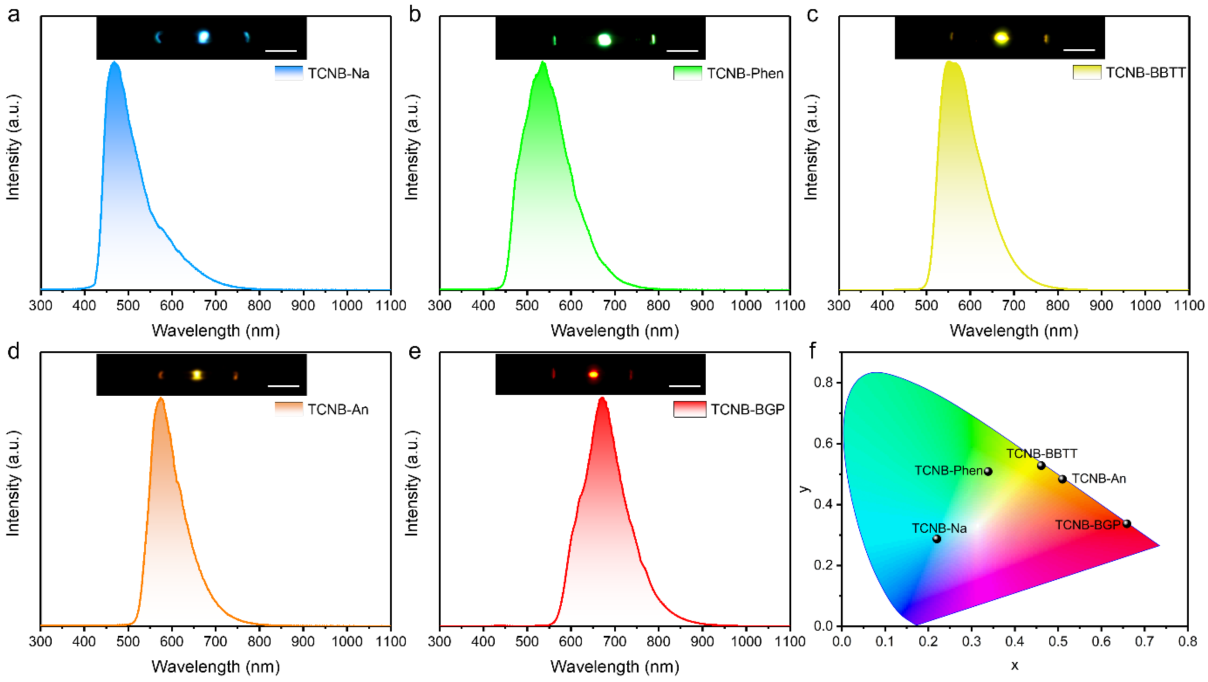

Figure S23. (a-e) Spatially resolved PL spectra of different types of CT cocrystals. Inset: FM images of CT microrods excited using a laser beam (λ = 395 nm). Scale bars: 50 μm. (f) The corresponding CIE chromaticity diagram.

Table S1. Crystal data and structure refinement for TBA and TBB.

Name	TBA	TBB
CCDC Number	1103093	1964761
Empirical formula	$C_{24}H_{20}N_4$	$C_{32}H_{14}N_4$
Formula weight	356.38	454.48
Crystal system	Monoclinic	Orthorhombic
Space group	P 2_1/a	P n a 21
a (Å)	9.441(2)	9.6471(7)
b (Å)	12.650(4)	31.514(2)
c (Å)	7.299(1)	7.1332(5)
α (°)	90	90
β (°)	93.11	90
γ (°)	90	90
Cell Volume (Å^3)	870.426	2168.63
Z, Z'	2, 0	4, 0
R-Factor (%)	3.6	3.54

Table S2. Attachment energies of various crystal facets (*hkl*) of TBA crystal calculated by using Materials Studio.

hkl	d_{hkl} (Å)	E_{att} (kcal mol^{-1})	Total facet area (%)
{110}	7.56	-47.44	55.54
{001}	7.29	-46.72	32.32
{020}	6.33	-60.92	8.90
{011}	6.32	-65.84	3.24
{11-1}	5.36	-80.42	-

Table S3. Attachment energies of various crystal facets (*hkl*) of TBB crystal calculated by using Materials Studio.

hkl	d_{hkl} (Å)	E_{att} (kcal mol^{-1})	Total facet area (%)
{020}	15.76	-48.99	46.71
{110}	9.22	-97.57	26.02
{120}	8.23	-98.24	3.89
{130}	7.11	-109.65	-
{011}	6.96	-119.35	11.69
{01-1}	6.96	-119.35	11.69
{031}	5.90	-139.20	-
{03-1}	5.90	-139.20	-
{111}	5.64	-161.62	-
{11-1}	5.64	-161.62	-

3. References

[1] C. Wang, H. Dong, L. Jiang, W. Hu, *Chem. Soc. Rev.* **2018**, 47, 422–500.

[2] D. Zhang, J. De, Y. Lei, H. Fu, *Nat. Commun.* **2021**, *12*, 1838.

[3] T. Lu, Q. Chen, *J. Comput. Chem.* **2022**, *43*, 539–555.

[4] V. Choudhary, A. Bhatt, D. Dash, N. Sharma, *J. Comput. Chem.* **2019**, *40*, 2354–2363.

[5] D. Robinson, *J. Chem. Theory Comput.* **2018**, *14*, 5303–5309.
